# The Relationship of Vaginal Symptoms and Cervical Inflammation Severity with Cytological Abnormalities and HPV Positivity: A Prospective Observational Study

**DOI:** 10.3390/biomedicines14061384

**Published:** 2026-06-19

**Authors:** Alihan Tigli, Rulin Deniz, Toros Taskin, Guzide Ece Akinci, Sultan Deniz Altindag, Nazli Sener, Yasemin Ercan Degirmenci, Sefer Ustebay, Muhammet Bora Uzuner, Erdem Gurkan, Oguzhan Karakoc, Yakup Baykus

**Affiliations:** 1 Department of Obstetrics and Gynecology, Faculty of Medicine, Bandırma Onyedi Eylül University, 10200 Bandırma, Türkiye; atigli@bandirma.edu.tr (A.T.); rdeniz@bandirma.edu.tr (R.D.); nsener@bandirma.edu.tr (N.S.); ydegirmenci@bandirma.edu.tr (Y.E.D.); 2 Department of Medical Pathology, Faculty of Medicine, Bandırma Onyedi Eylül University, 10200 Bandırma, Türkiye; ttaskin@bandirma.edu.tr (T.T.); saltindag@bandirma.edu.tr (S.D.A.); 3 Gynecology and Obstetrics Clinic, Şehit Prof. Dr. İlhan Varank Sancaktepe Training and Research Hospital, 34785 Istanbul, Türkiye; geceakinci@gmail.com; 4 Department of Pediatrics, Faculty of Medicine, Bandırma Onyedi Eylül University, 10200 Bandırma, Türkiye; sustebay@bandirma.edu.tr; 5 Department of Anatomy, Faculty of Medicine, Bandırma Onyedi Eylül University, 10200 Bandirma, Türkiye; muzuner@bandirma.edu.tr; 6 Gynecology and Obstetrics Clinic, Bandırma Training and Research Hospital, 10200 Bandirma, Türkiye; mderdem.gurkan@bandirma.edu.tr (E.G.); oguzhankarakoc1986@hotmail.com (O.K.)

**Keywords:** vaginal symptoms, cervical inflammation, Pap smear, human papillomavirus, cervical cytology, Candida

## Abstract

**Background/Objectives**: This study aimed to evaluate the association between the clinical parameters of vaginal infection—specifically the presence, type, number of concurrent symptoms, and recurrence frequency—and cervical cytology findings, including inflammation severity, Candida, bacterial vaginosis, cellular abnormalities, and Human Papillomavirus (HPV) positivity. **Methods**: This prospective, cross-sectional, observational study included 458 women attending our gynecology outpatient clinic for Pap smear screening. Vaginal symptoms were documented through face-to-face interviews using a structured data collection form. Cervical samples were evaluated via liquid-based cytology by a single, experienced cytopathologist, who was blinded to the clinical data; cellular abnormalities, the degree of inflammation and cytomorphological findings indicative of infection were reported. HPV analysis was performed on the 218 women for whom results were available. Chi-square and trend chi-square tests were used in the statistical analysis. **Results**: No significant association was found between the clinical parameters of vaginal symptoms—specifically presence, concurrency, and recurrence frequency—and cytological abnormalities, HPV positivity and bacterial vaginosis (*p* > 0.05). In contrast, the prevalence of moderate-to-severe inflammation was significantly higher in women with vaginal discharge and a greater symptom burden (*p* < 0.05). Pruritus, dysuria, and vaginal burning were significantly associated with Candida positivity (*p* < 0.05). However, no significant association was found between the severity of cervical inflammation and abnormal cytology or HPV positivity (*p* > 0.05). In multivariable logistic regression analyses, neither symptom burden nor cervical inflammation severity was independently associated with abnormal cytology or HPV positivity. **Conclusions**: Our findings indicate that vaginal symptoms and the severity of cervical inflammation may not serve as definitive or independent discriminatory markers for cellular abnormalities or HPV positivity in this context. Nevertheless, specific symptom patterns may assist clinicians in evaluating localized infectious processes. Consequently, while standard cytological and molecular protocols remain essential for oncogenic screening, evaluating the overall symptom burden provides clinicians with a valuable framework for identifying benign dysbiotic and inflammatory processes. These findings remained consistent after adjustment for major clinical confounders.

## 1. Introduction

Lower genital tract complaints represent one of the primary reasons for consultations in gynecology and obstetrics clinics. Symptoms such as vaginal discharge, pruritus, burning, malodor, dysuria, dyspareunia, and lower abdominal pain are highly prevalent among women of reproductive age and are frequently clinical indicators of vaginal infection [1]. However, the etiology of these complaints is remarkably heterogeneous; while symptoms may be associated with infectious conditions such as bacterial vaginosis or vulvovaginal candidiasis, they may also arise from physiological discharge, local irritation, hormonal fluctuations, or other non-infectious processes [2]. Consequently, clinical assessment based solely on symptomatology often proves insufficient for precise pathological identification. Furthermore, the low specificity of patient-reported symptoms makes it difficult to establish a reliable microbiological diagnosis [3,4]. While the type, number, and frequency of symptoms are clinically significant, and specific symptom patterns may correlate with the degree of cervical inflammation, Candida colonization, bacterial vaginosis, Human Papillomavirus (HPV) positivity, and cytological abnormalities, it is emphasized that these associations vary considerably across different clinical parameters [5,6,7].

While the primary objective of the cervical smear is to screen for pre-invasive lesions, it also yields valuable insights into inflammatory changes and cytological markers suggestive of infectious agents, alongside cellular abnormalities [8]. However, the clinical significance of inflammation detected in a smear is not always clear. While cytological inflammation may reflect an active infection, it may also be associated with local irritation, transient reactive changes, hormonal effects or sampling variations [9,10]. Consequently, a holistic approach is required when interpreting smear results that indicate inflammation, considering the patient’s clinical profile and laboratory findings [11].

The available data in the literature indicate that the multifaceted relationship between lower genital tract symptoms, the severity of inflammation detected in smear tests, HPV positivity and cytological abnormalities has not yet been fully clarified. Although the presence of symptoms is frequently interpreted in clinical practice as indicative of infection, the extent to which these complaints correlate with specific microbiological findings—such as the presence of Candida or bacterial vaginosis—and cytological results remains a matter of debate [12]. In particular, the uncertainty and conflicting findings regarding the relationship between the severity of cervical inflammation and abnormal cytology and HPV positivity raise the question of how inflammatory cellular changes should be interpreted in the context of neoplastic risk [13,14,15,16].

As the role of symptoms and inflammatory findings in predicting neoplastic processes has not yet been fully elucidated, clinical data must be examined using a holistic approach. Accordingly, the aim of our study is to assess the presence, type, number and frequency of vaginal infection symptoms in women attending the gynecology outpatient clinic; to investigate the relationship between these symptoms and the severity of inflammation detected in cervical smears, HPV positivity, and the morphological findings and cytological results of Candida and bacterial vaginosis; and to examine the relationship between the severity of inflammation in the smear and abnormal cytology and HPV positivity. Unlike prior studies that frequently evaluate vaginal symptoms as merely present or absent, the present study contributes a novel perspective to the literature by comprehensively analyzing the “symptom burden” (specifically, the number of concurrent symptoms and their frequency of recurrence) in conjunction with semi-quantitative inflammation scoring. Our study is based on the hypothesis that these symptoms and the severity of inflammation demonstrate a more consistent correlation with specific microbiological and localized reactive processes, rather than with cellular risk factors such as abnormal cytology and HPV positivity.

## 2. Materials and Methods

### 2.1. Study Design and Sampling

**Figure 1:**
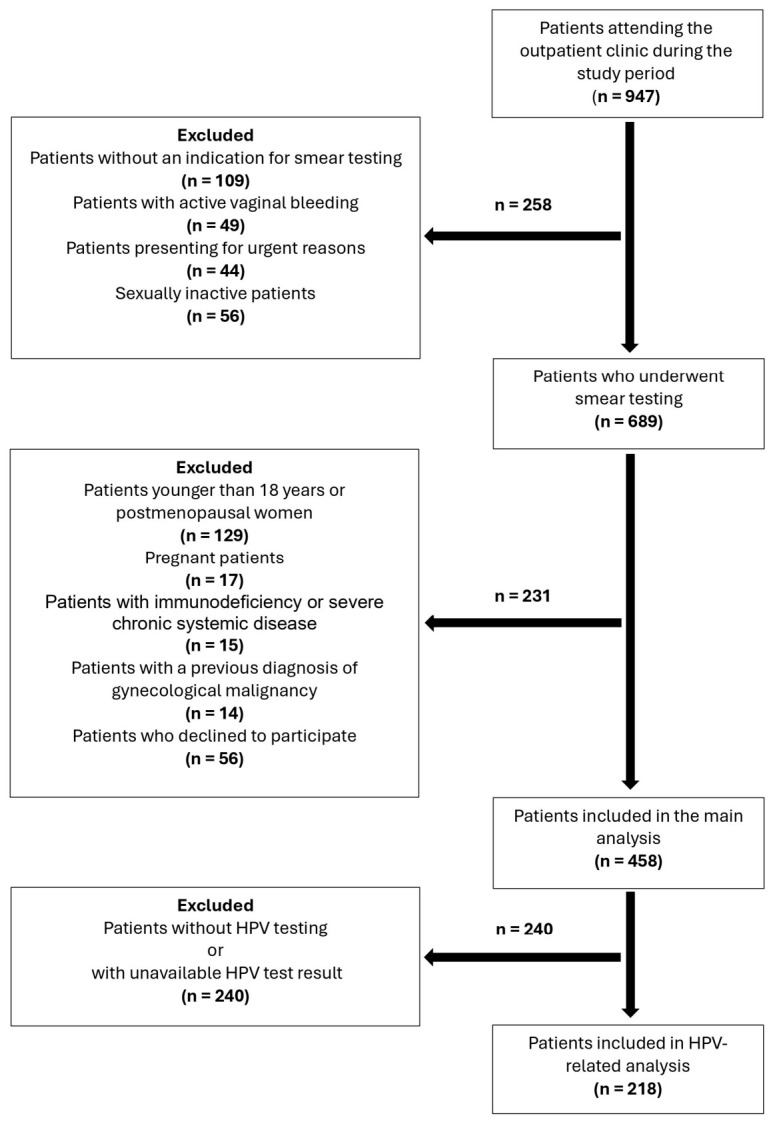
Flow chart of study design.

This study was conducted as a single-center, cross-sectional observational study with prospective patient recruitment and real-time data collection. The study included women who attended the Obstetrics and Gynecology Outpatient Clinic at Bandırma Onyedi Eylül University Hospital between November 2025 and April 2026 and underwent a cervical smear test. The sample size for the study was determined using the G*Power 3.1.9.7 software (Heinrich-Heine-Universität Düsseldorf, Düsseldorf, Germany), based on data from the literature by Konyalıoğlu and Yılmaz (effect size Cohen’s w = 0.461) [17,18,19]. It was estimated that a minimum of 248 participants would be required to achieve 95% power and a 5% Type 1 error rate (α = 0.05); to account for potential data losses, an additional 30% of cases were included as a buffer, with the aim of involving at least 322 women in the study. Following the application of the study’s inclusion and exclusion criteria, a total of 458 women were assessed within the scope of the study. Analyses related to HPV were conducted on 218 women aged 30 and over who had undergone HPV testing as part of the national cancer screening program in our country and for whom data was available (Figure 1).

### 2.2. Data Collection Process and Variables

The research data were collected through the evaluation of clinical information obtained via face-to-face interviews, smear samples taken during vaginal examinations, and HPV screening results. During the interviews, participants’ sociodemographic and clinical characteristics (age, educational level, income status, presence of chronic diseases, number of births and miscarriages, contraceptive methods, etc.), as well as lifestyle factors, were recorded in detail. Patients’ symptomatic conditions associated with vaginal infection (discharge, malodor, pruritus, pelvic pain, dyspareunia, dysuria and vaginal burning) were prospectively recorded using a structured data collection form developed by the researchers considering the current literature data. To reflect the symptom burden more objectively, the number of concurrent symptoms and the frequency of recurrence were also included in the analyses. This systematic approach aimed to obtain patients’ subjective reports within a standardized framework and to minimize data loss. Following clinical assessment, cervical cytological samples were collected from the transformation zone using a brush after speculum examination, transferred to a liquid-based cytology solution using the BD SurePath system (Becton, Dickinson and Company, Franklin Lakes, NJ, USA), and examined in accordance with standard laboratory protocols. HPV testing was performed as part of the national cervical cancer screening program using the cobas HPV Test (Roche Molecular Systems, Pleasanton, CA, USA), a validated real-time PCR assay to detect high-risk HPV (HR-HPV) DNA. The assay specifically identifies 14 high-risk genotypes (16, 18, 31, 33, 35, 39, 45, 51, 52, 56, 58, 59, 66, and 68), with concurrent genotyping for HPV 16 and HPV 18.

### 2.3. Cytological and Inflammatory Assessment

**Figure 2:**
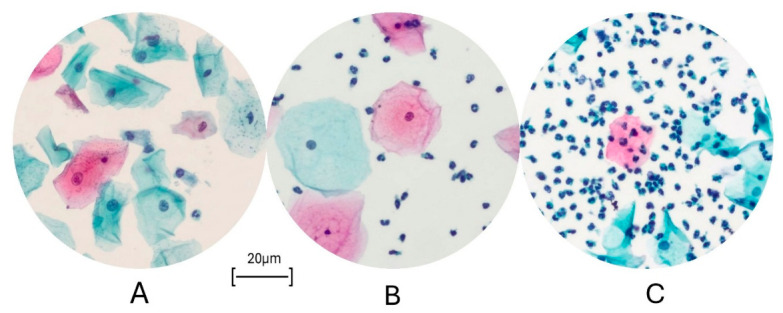
Assessment of inflammation in smear specimens (400×). (**A**) None/minimal inflammation (<10 leukocytes/HPF), (**B**) moderate inflammation (10–100 leukocytes/HPF), (**C**) severe inflammation (>100 leukocytes/HPF).

All cervical cytology slides and inflammation scoring were analyzed and reported by a single, experienced cytopathologist, who was blinded to the clinical data, in accordance with the Bethesda System criteria, with the aim of eliminating potential inter-observer variability and maximizing internal consistency [20]. The presence of Candida in smear samples and cytomorphological findings consistent with bacterial vaginosis were also considered [9]. It should be noted that the presence of Candida and bacterial vaginosis was evaluated solely based on cytomorphological features in the Pap smear; confirmatory microbiological tests such as culture, nucleic acid amplification tests (NAAT), Amsel criteria, or Nugent scoring were not performed. The severity of inflammation was scored semi-quantitatively based on the leukocyte density in the high-power field (400 × HPF); accordingly, <10 leukocytes/HPF was classified as “minimal/absent”, 10–100 leukocytes/HPF as “moderate”, and >100 leukocytes/HPF as “severe” inflammation (Figure 2). This semi-quantitative scoring system is a locally developed approach routinely used in our institution’s pathology laboratory, rather than an internationally validated guideline.

### 2.4. Data Analysis

Statistical analysis of the data was performed using IBM SPSS Statistics for Windows, version 26.0 (IBM Corp., Armonk, NY, USA). Continuous variables are presented as mean and standard deviation, while categorical variables are presented as frequency and percentage. The Pearson chi-square test was used to compare the associations between symptoms of vaginal infection and cervical cytopathology and HPV findings. For analyses involving ordinal data, such as the number or frequency of symptoms, the trend chi-square test was applied, and a significance level of *p* < 0.05 was adopted for all tests.

The primary endpoints were predefined as abnormal cytology and HPV positivity. Cervical inflammation severity, Candida positivity, and bacterial vaginosis were considered secondary exploratory outcomes. Because multiple comparisons were performed across several symptoms and outcomes, the Benjamini–Hochberg false discovery rate procedure was applied to exploratory analyses to reduce the risk of type I error. Findings from secondary exploratory analyses were interpreted cautiously.

In addition to Pearson chi-square and chi-square test for trend analyses, multivariable binary logistic regression analyses were performed to evaluate whether vaginal symptom parameters and cervical inflammation severity were independently associated with abnormal cytology and HPV positivity. Abnormal cytology and HPV positivity were entered as separate binary dependent variables. The models were adjusted for age, smoking status, parity, vaginal discharge, symptom recurrence frequency, Candida positivity, bacterial vaginosis, cervical inflammation severity, and symptom burden. Adjusted odds ratios (aORs) with 95% confidence intervals (CIs) were reported. Model fit was assessed using the Hosmer–Lemeshow goodness-of-fit test. The HPV positivity model was performed only among participants with available HPV results.

## 3. Results

### 3.1. Characteristic Features and Distribution of Symptoms

The mean age of the 458 women included in the study was 38.18 ± 9.41 years; a detailed breakdown of their sociodemographic and clinical characteristics is presented in Table 1. The vast majority of participants (84.5%) had no chronic illnesses, and 78.2% engaged in regular physical activity. Regarding contraceptive use, the barrier method was the most preferred method (32.5%), while 58.1% of women were not using any form of contraception.

When the distribution of symptoms of vaginal infection was assessed, it was determined that the most frequently reported complaint during clinical consultations was vaginal discharge (57.5%). This was followed, in order, by lower abdominal pain (37.3%), pruritus (24.8%), malodor (24.2%) and dyspareunia (22.2%); while complaints of vaginal burning (17.9%) and dysuria (13.5%) were reported at lower rates compared to other symptoms (Table 2).

### 3.2. The Relationship Between Vaginal Symptoms and Cytological, Inflammatory and Microbiological Findings

When the relationship between symptoms of vaginal infection and cervical cytology, HPV and findings of infection was examined, no statistically significant difference was found in terms of abnormal cytological findings, bacterial vaginosis (*n* = 458) and HPV positivity (*n* = 218) for all symptom types (discharge, malodor, pruritus, lower abdominal pain, dyspareunia, dysuria and vaginal burning) (Table 3). Detailed evaluation of the cervical cytology results according to the Bethesda system revealed that the entirely benign Cytology (−) group (*n* = 398) consisted exclusively of NILM cases, as ASC-US cases were deliberately excluded from the study to prevent borderline confounding. The Cytology (+) group (*n* = 60), which was strictly defined as LSIL and above for clinical significance, comprised 46 LSIL, 2 HSIL, 8 ASC-H, 3 AGC, and 1 SCC cases. Among the 218 women with available HPV data, a total of 32 cases were positive for high-risk HPV. Genotype distribution of these positive cases revealed that 12 women were positive for HPV 16, 3 for HPV 18, and 17 for other high-risk genotypes. However, the incidence of moderate-to-severe inflammation was significantly higher in women with discharge symptoms than in those without (*p* = 0.001). When specific microbiological findings were evaluated, it was found that the rate of Candida positivity was significantly higher in cases with complaints of pruritus (*p* < 0.001), dysuria (*p* < 0.001) and vaginal burning (*p* = 0.001) compared to other women (Table 3).

When the relationship between the number of symptoms, frequency of recurrence and clinical findings was assessed, an increased symptom burden was not significantly associated with abnormal cytology, bacterial vaginosis or HPV positivity (Table 4). Conversely, it was found that as the number of concurrent symptoms increased, the rate of minimal inflammation decreased, while the rates of moderate and severe inflammation increased significantly (*p* = 0.005). A similar trend was observed regarding symptom frequency: increased symptom recurrence was significantly associated with a higher rate of severe inflammation (*p* = 0.043). Furthermore, it was determined that the rate of Candida positivity also increased significantly as the number of symptoms increased (*p* = 0.004) (Table 4).

Because multiple symptom–outcome comparisons were performed, the Benjamini–Hochberg false discovery rate procedure was applied to exploratory analyses. After FDR correction, the associations between vaginal discharge and cervical inflammation, between pruritus, dysuria, vaginal burning and Candida positivity, and between symptom burden and both cervical inflammation and Candida positivity remained statistically significant. However, the association between symptom recurrence frequency and cervical inflammation did not remain robust after correction and was therefore interpreted cautiously.

### 3.3. The Relationship Between the Severity of Cervical Inflammation and Cytological Abnormalities and HPV

When examining the relationship between the severity of cervical inflammation, cellular abnormalities, and HPV carriage, a numerical trend toward higher rates of abnormal cytology with increasing inflammatory severity was observed; however, this association did not reach statistical significance (*p* = 0.159). Similarly, no significant difference was found between the degree of cervical inflammation and HPV positivity (*p* = 0.342) (Table 5).

### 3.4. Multivariable Logistic Regression Analysis for Abnormal Cytology and HPV Positivity

In the abnormal cytology model, none of the evaluated variables showed an independent association with abnormal cytology after adjustment for potential confounders. Similarly, in the HPV subgroup, no independent association was observed between HPV positivity and vaginal discharge, symptom burden, Candida positivity, bacterial vaginosis, or cervical inflammation severity. Model calibration was acceptable according to the Hosmer–Lemeshow goodness-of-fit test for both the abnormal cytology model and the HPV positivity model (Table 6).

## 4. Discussion

In this study, women presenting with symptoms of vaginal infection were compared with asymptomatic individuals to comprehensively evaluate the relationship between vaginal symptom burden and cervical inflammation, alongside cytopathological, microbiological, and HPV parameters. While the primacy of cytological and molecular testing over symptom-based screening is clinically well-established, our study aims to build upon this knowledge by granularly analyzing the symptom burden in conjunction with cervical inflammation scores. By demonstrating that an increased symptom burden strongly correlates with local reactive inflammation and specific microbial patterns rather than oncogenic markers, this study provides a clinical framework that delineates benign dysbiotic processes from actual neoplastic risk. The findings obtained in our study are discussed below under the following subheadings, in conjunction with data from the current literature.

### 4.1. The Association Between Vaginal Symptoms and Abnormal Cytology and HPV Positivity

The findings of our study suggest that the type, number and frequency of lower genital tract symptoms did not exhibit a statistically significant association with cervical cytological abnormalities and HPV positivity. However, due to the limited sample size of the HPV subset and the lack of adjustment for major confounding variables, the absence of statistical significance should not be definitively interpreted as a complete lack of association. Although complaints such as vaginal discharge, malodor, burning or dyspareunia are among the most common reasons for gynecological consultations, our current data support the hypothesis that these symptoms reflect local inflammatory and dysbiotic processes rather than cervical intraepithelial neoplasia (CIN) or HPV positivity [21]. Although it has been reported in the literature that certain forms of dysbiosis—such as moderate or severe aerobic vaginitis—may be associated with major cervical abnormalities or high-risk HPV [22], this suggests that subjective symptoms reported by patients should not be interpreted as a direct indicator of oncogenic risk [23]. Similarly, the fact that some reported correlations between bacterial vaginosis and high-risk HPV weaken or disappear when adjusted for confounding behavioral factors [24,25] suggests that symptoms may be associated not directly with dysplasia, but primarily with lifestyle factors or transient changes in the microbiome. Given that HPV-related cervical carcinogenesis is a slow-progressing, subclinical process—typically remaining asymptomatic in its early stages—it is imperative to recognize that women may harbor high-risk HPV genotypes even in the absence of overt vaginal symptoms [26]. The findings we have obtained in this regard reinforce the view that cervical screening approaches should be based on standard cytological and molecular methods rather than on the presence of vaginal symptoms [27]. Importantly, the multivariable logistic regression analyses supported the findings of the univariate comparisons. After adjustment for age, smoking status, parity, vaginal discharge, symptom recurrence frequency, Candida positivity, bacterial vaginosis, cervical inflammation severity, and symptom burden, neither symptom-related parameters nor cervical inflammation severity were independently associated with abnormal cytology or HPV positivity. These findings suggest that the absence of significant associations in the univariate analyses was not merely attributable to the lack of adjustment for major clinical confounders. Rather, vaginal symptoms and smear-based inflammatory findings appear to be more closely related to localized infectious or reactive processes than to cytological abnormality or HPV positivity.

### 4.2. The Relationship Between Vaginal Symptoms and Cervical Inflammation

The observation that vaginal discharge exhibits a more robust correlation with the severity of inflammation in the smear-detected inflammation compared to other symptoms aligns with the existing literature, which underscores the intricate interaction between alterations in the vaginal microbiome and the host’s local immune response. Vaginal discharge often reflects a shift from a healthy microenvironment dominated by Lactobacillus to a dysbiotic environment in which anaerobes predominate; these microbial changes increase local pro-inflammatory cytokine production, thereby predisposing to mucosal irritation [28,29]. The fact that increases in the number of concurrent symptoms and recurrence frequency are associated with more pronounced inflammation also supports this mechanism [30,31]. Conversely, the fact that symptoms such as malodor, dyspareunia or a burning sensation do not show a significant correlation with the severity of cervical inflammation suggests that these complaints may stem from more heterogeneous mechanisms, such as hormonal fluctuations, local irritation or non-infectious conditions [32].

Although several symptom–outcome associations were observed in unadjusted analyses, multiple testing correction was applied to reduce the risk of false-positive findings. After FDR correction, the most robust secondary findings were the associations of Candida positivity with pruritus, dysuria, vaginal burning, and symptom burden, as well as the associations of cervical inflammation with vaginal discharge and symptom burden. In contrast, the association between symptom recurrence frequency and cervical inflammation was weaker and should be interpreted as exploratory.

### 4.3. The Relationship Between Vaginal Symptoms and Candida and Bacterial Vaginosis

The finding that symptoms of pruritus, dysuria and vaginal burning are strongly associated with the presence of Candida supports the established pathophysiology of vulvovaginal candidiasis [33,34]. As the host response in vulvovaginal candidiasis is characterized by profound local inflammatory signaling, symptoms such as pruritus and burning naturally demonstrate a closer correlation with this microbiological profile [35]. The fact that the relationship between symptom frequency and Candida positivity is not strictly linear can be explained by the complex nature of recurrent vulvovaginal candidiasis, which is influenced by variations in fungal load, host response and microbial interactions [36,37]. On the other hand, the discrepancy observed in our study between vaginal symptom profiles and the findings of bacterial vaginosis detected in smear tests is striking. Several studies indicate that, in clinical practice, a diagnosis of bacterial vaginosis based solely on symptoms such as discharge and malodor lacks adequate diagnostic sensitivity and specificity [38]. The observed discrepancy is fundamentally linked to the frequently asymptomatic nature of bacterial vaginosis, and that the absence of clinical symptoms does not preclude the presence of a dysbiotic microbial profile [39,40]. Bacterial vaginosis is fundamentally characterized by a profound ecological shift from a Lactobacillus-dominant state to a polymicrobial dysbiosis. In this context, *Gardnerella vaginalis* acts as a keystone pathogen, initiating the pathogenic process through the formation of a robust multi-species biofilm on the vaginal epithelium. This biofilm not only facilitates the adherence and proliferation of other strictly anaerobic bacteria but also provides a protective niche against host immune responses. Consequently, the prominent presence of G. vaginalis and its associated biofilm are central to both the clinical manifestation of the typical malodorous discharge and the frequently recurrent, treatment-resistant nature of bacterial vaginosis. Furthermore, the possibility that smear-based morphological examination may not fully reflect the functional and metabolic dynamics of the vaginal ecosystem underscores the need to assess symptom burden alongside advanced molecular methods (such as NAAT or NGS) to definitively identify specific etiological agents (e.g., Trichomonas vaginalis, Chlamydia trachomatis, Neisseria gonorrhoeae, and specific *Candida* and *Gardnerella* species).

### 4.4. The Relationship Between the Severity of Inflammation and HPV Positivity and Abnormal Cytology

The lack of significant association between severity of cervical inflammation and oncogenic indicators, such as abnormal cytology or HPV positivity, underscores the clinical need to differentiate benign reactive changes from neoplastic transformations. Chronic inflammation caused by infections or local irritants can lead to atypical cellular changes that mimic precancerous lesions, thereby creating difficulties in cytological assessment [41,42,43]. Although it has been suggested that severe inflammation may disrupt the epithelial barrier and thereby create a conducive environment for HPV oncogenesis [44,45,46], our findings support the premise that inflammation alone cannot be used as an indicator of HPV infection or neoplastic transformation. Inflammation in the smear more likely reflects a transient local immune response, irritation or dysbiosis.

### 4.5. Strengths and Limitations of the Study

Our study evaluated the diversity of symptoms, the number of concurrent conditions and the frequency of recurrence by comparing symptomatic and asymptomatic women within the same sample; it analyzed cervical cytopathology, the severity of inflammation, specific findings of infection and HPV positivity in an integrated manner. This multifaceted approach contributes to the objective differentiation of infectious, local inflammatory and neoplastic processes. However, certain limitations should be borne in mind. The reliance on patient-reported symptoms may have introduced recall bias. The diagnosis of Candida and bacterial vaginosis was based solely on Pap smear cytomorphology without confirmation by gold-standard microbiological methods (e.g., culture, NAAT, Amsel criteria, or wet mount microscopy). Although this is a major limitation that may fail to fully reflect all changes in the vaginal microbiota—meaning these outcomes should be interpreted as cytological markers rather than definitive diagnoses—the primary aim of our study was not the definitive detection of pathogens, but rather to evaluate the clinical implications of routine inflammatory findings. Additionally, although blinding of the cytopathologist minimized clinical bias, the assessment of all samples by a single pathologist without an interobserver agreement analysis limits the reproducibility of the semi-quantitative inflammation scoring and cytological evaluations. Furthermore, the reliance on a locally developed, non-validated scoring system for inflammation severity limits the external generalizability of these findings. Our large sample size (*n* = 458) and 95% statistical power support the clinical validity of the findings for the overall cohort; however, the HPV-related analyses were conducted on a substantially smaller subset (*n* = 218), which reduces the statistical power, especially for subgroup comparisons. Furthermore, the restriction of HPV analyses to women ≥ 30 years in accordance with national screening guidelines introduces an inevitable selection bias, meaning our HPV-related findings may not be fully generalizable to younger populations. Moreover, the lack of data on critical confounding factors—such as detailed sexual history (including the number of sexual partners, age at first intercourse, and history of previous sexually transmitted infections), previous HPV vaccination status, and history of abnormal smears—limits the generalizability and interpretability of our HPV-related findings. Although multivariable logistic regression analyses were performed to adjust for clinically relevant confounders, the number of HPV-positive cases was limited, which may have resulted in wide confidence intervals for some estimates. Therefore, the adjusted HPV findings should be interpreted cautiously and confirmed in larger cohorts. The cross-sectional design does not allow for inferences of causality, while the single-center design limits generalizability.

## 5. Conclusions

Our study found that the type, number and frequency of vaginal symptoms did not present as definitive predictors of cervical cytological abnormalities or HPV positivity in this specific cohort; moreover, this conclusion should be interpreted with caution given the small HPV sample size and the presence of unmeasured confounders. Nevertheless, it was determined that certain symptom patterns may contribute to the clinical assessment of local infectious and inflammatory processes. Vaginal discharge and an increased symptom burden were found to be significantly associated with cervical inflammation, while pruritus and burning complaints were significantly associated with the presence of Candida. It was also observed that the severity of cervical inflammation alone may not be sufficient for predicting abnormal cytology and HPV positivity without further molecular confirmation. Considering these findings, while it is reaffirmed that cervical screening must rely on standard cytological and molecular methods rather than subjective symptoms, our study highlights that comprehensively evaluating the symptom burden provides a valuable clinical framework for identifying benign dysbiotic and inflammatory processes. In future studies, a more detailed examination of the symptom–dysbiosis relationship using advanced molecular microbiology methods (e.g., NAAT and NGS to definitively detect the aforementioned specific pathogens) alongside multicenter designs will contribute to the literature.

## Figures and Tables

**Table 1:** Distribution of sociodemographic and clinical characteristics (*n* = 458).

Sociodemographic and Clinical Characteristics	(*n*)	(%)
**Age (Years)**		
Mean ± SD: 38.18 ± 9.41, min: 18, max: 54 years		
**Cytology results**		
Benign	398	86.9
AGC	3	0.7
ASC-H	8	1.7
LSIL	46	10.0
HSIL	2	0.4
SCC	1	0.2
**HPV results**		
Positive	32	7.0
HPV 16	12	2.6
HPV 18	3	0.7
Other HR-HPV	17	3.7
Negative	186	40.6
Age-ineligible (<30 years)	101	22.1
Declined HPV testing	139	30.3
**Level of education**		
Primary school and below	156	34.1
High school	135	29.5
University and above	167	36.5
**Income level**		
Income less than expenditure	91	19.9
Income equals expenditure	304	66.4
Income exceeds expenditure	63	13.8
**Chronic illness**		
No	387	84.5
Yes	71	15.5
**Smoking status**		
No	279	60.9
Yes	179	39.1
**Alcohol consumption**		
No	385	84.1
Yes	73	15.9
**Physical activity**		
Yes	358	78.2
No	100	21.8
**Number of births**		
0	116	25.3
1	107	23.4
2	173	37.8
≥3	62	13.5
**Number of miscarriages**		
0	346	75.5
1	79	17.2
≥2	33	7.2
**Number of living children**		
0	116	25.3
1	110	24.0
2	171	37.3
≥3	61	13.3
**Contraceptive method used**		
None	266	58.1
Vaginal douche	3	0.7
IUD	40	8.7
Condom	149	32.5

Note: *n*, number; %, percentage; SD, standard deviation; min, minimum; max, maximum; IUD, intrauterine device; AGC, atypical glandular cells; ASC-H, atypical squamous cells, cannot exclude HSIL; LSIL, low-grade squamous intraepithelial lesion; HSIL, high-grade squamous intraepithelial lesion; SCC, squamous cell carcinoma; HR-HPV, high-risk human papillomavirus.

**Table 2:** Distribution of vaginal symptoms.

Symptoms	(*n*)	(%)
**Vaginal discharge**		
No	195	42.5
Yes	264	57.5
**Vaginal Malodor**		
No	348	75.8
Yes	111	24.2
**Vaginal Pruritus**		
No	345	75.2
Yes	114	24.8
**Groin pain**		
No	288	62.7
Yes	171	37.3
**Dyspareunia**		
No	357	77.8
Yes	102	22.2
**Dysuria**		
No	397	86.5
Yes	62	13.5
**Vaginal burning**		
No	377	82.1
Yes	82	17.9

Note: *n*, number; %, percentage.

**Table 3:** Comparison of cervical cytology, severity of inflammation, cytomorphological findings indicative of infection (*n* = 458), and HPV positivity (*n* = 218) by symptom type.

Symptoms	Cytology		Inflammation			HPV		Candida		BV	
	(−) %(*n*)	(+) %(*n*)	Minimal %(*n*)	Moderate %(*n*)	Severe %(*n*)	(−) %(*n*)	(+) %(*n*)	(−) %(*n*)	(+) %(*n*)	(−) %(*n*)	(+) %(*n*)
**Vaginal discharge**											
No	87.1(169)	12.9(25)	39.7(77)	49.5(96)	10.8(21)	84.0(89)	16.0(17)	96.9(188)	3.1(6)	95.4(185)	4.6(9)
Yes	86.7(229)	13.3(35)	26.1(69)	53.0(140)	20.8(55)	86.6(97)	13.4(15)	93.9(248)	6.1(16)	97.0(256)	3.0(8)
Test value	0.014		13.648			0.304		2.154	0.810		
*p*	0.907		**0.001**			0.581		0.142	0.386		
**Vaginal Malodor**											
No	87.3(303)	12.7(44)	34.6(120)	49.6(172)	15.9(55)	84.0(147)	16.0(28)	96.0(333)	4.0(14)	96.3(334)	3.7(13)
Yes	85.6(95)	14.4(16)	23.4(26)	57.7(64)	18.9(21)	90.7(39)	9.3(4)	92.8(103)	7.2(8)	96.4(107)	3.6(4)
Test value	0.222		4.830			1.236		1.851	0.005		
*p*	0.637		0.089			0.266		0.174	0.945		
**Vaginal Pruritus**											
No	86.6(298)	13.4(46)	33.7(116)	50.6(174)	15.7(54)	84.2(139)	15.8(26)	97.4(335)	2.6(9)	96.5(332)	3.5(12)
Yes	87.7(100)	12.3(14)	26.3(30)	54.4(62)	19.3(22)	88.7(47)	11.3(6)	88.6(101)	11.4(13)	95.6(109)	4.4(5)
Test value			2.382			0.631		14.459		0.193	
*p*			0.304			0.427		**<0.001**		0.660	
**Groin pain**											
No	87.5(251)	12.5(36)	35.5(102)	48.1(138)	16.4(47)	83.8(109)	16.2(21)	95.8(275)	4.2(12)	96.9(278)	3.1(9)
Yes	86.0(147)	14.0(24)	25.7(44)	57.3(98)	17.0(29)	87.5(77)	12.5(11)	94.2(161)	5.8(10)	95.3(163)	4.7(8)
Test value	0.209		5.026			0.559		0.651		0.713	
*p*	0.647		0.081			0.455		0.420		0.398	
**Dyspareunia**											
No	86.8(309)	13.2(47)	33.1(118)	50.6(180)	16.3(58)	86.0(147)	14.0(24)	95.8(341)	4.2(15)	96.1(342)	3.9(14)
Yes	87.3(89)	12.7(13)	27.5(28)	54.9(56)	17.6(18)	83.0(39)	17.0(8)	93.1(95)	6.9(7)	97.1(99)	2.9(3)
Test value	0.015		1.184			0.262		1.217		0.218	
*p*	0.904		0.553			0.608		0.270		0.641	
**Dysuria**											
No	86.6(343)	13.4(53)	33.3(132)	51.3(203)	15.4(61)	85.3(162)	14.7(28)	96.7(383)	3.3(13)	96.7(383)	3.3(13)
Yes	88.7(55)	11.3(7)	22.6(14)	53.2(33)	24.2(15)	85.7(24)	14.3(4)	85.5(53)	14.5(9)	93.5(58)	6.5(4)
Test value	0.206		4.480			0.004		14.793		1.506	
*p*	0.650		0.106			0.950		**<0.001**		0.220	
**Vaginal burning**											
No	86.7(326)	13.3(50)	32.7(123)	50.5(190)	16.8(63)	85.7(150)	14.3(25)	96.8(364)	3.2(12)	96.3(362)	3.7(14)
Yes	87.8(72)	12.2(10)	28.0(23)	56.1(46)	15.9(13)	83.7(36)	16.3(7)	87.8(72)	12.2(10)	96.3(79)	3.7(3)
Test value	0.072		0.897			0.110		11.934		0.001	
*p*	0.789		0.639			0.741		**0.001**		0.978	

Note: HPV, human papillomavirus; BV, bacterial vaginosis; (−), negative; (+), positive; *n*, number; %, percentage; *p*, *p* value;Pearson chi-square test.

**Table 4:** Association between the burden of vaginal symptoms (number and frequency of recurrence) and cytopathological, cytomorphological findings indicative of infection (*n* = 458), and HPV positivity (*n* = 218).

	Cytology		Inflammation			HPV		Candida		BV	
	(−) %(*n*)	(+) %(*n*)	Minimal %(*n*)	Moderate %(*n*)	Severe %(*n*)	(−) %(*n*)	(+) %(*n*)	(−) %(*n*)	(+) %(*n*)	(−) %(*n*)	(+) %(*n*)
**Number of symptoms**											
0	87.4(97)	12.6(14)	46.8(52)	41.4(46)	11.7(13)	76.8(43)	23.2(13)	98.2(109)	1.8(2)	97.3(108)	2.7(3)
1–2	86.2(168)	13.8(27)	27.2(53)	53.8(105)	19.0(37)	88.9(80)	11.1(10)	97.4(190)	2.6(5)	95.9(187)	4.1(8)
3–4	88.3(98)	11.7(13)	29.7(33)	55.9(62)	14.4(16)	89.3(50)	10.7(6)	91.0(101)	9.0(10)	96.4(107)	3.6(4)
≥5	85.4(35)	14.6(6)	19.5(8)	56.1(23)	24.4(10)	81.3(13)	18.8(3)	87.8(36)	12.2(5)	95.1(39)	4.9(2)
Test value	0.002		18.324			5.087		13.518		0.559	
*p*	0.968		**0.005**			0.166		**0.004**		0.906	
**Frequency of symptoms**											
Low frequency (never/≤1 per year)	88.2(164)	11.8(22)	38.2(71)	49.5(92)	12.4(23)	78.9(71)	21.1(19)	97.3(181)	2.7(5)	96.8(180)	3.2(6)
Moderate frequency (2–11 times a year)	93.8(45)	6.3(3)	33.3(16)	54.2(26)	12.5(6)	89.3(25)	10.7(3)	83.3(40)	16.7(8)	95.8(46)	4.2(2)
High frequency (≥1 per month)	84.4(189)	15.6(35)	26.3(59)	52.7(118)	21.0(47)	90.0(90)	10.0(10)	96.0(215)	4.0(9)	96.0(215)	4.0(9)
Test value	1.386		9.864			5.073		16.895		0.209	
*p*	0.239		**0.043**			0.079		**<0.001**		0.901	

Note: HPV, human papillomavirus; BV, bacterial vaginosis; (−), negative; (+), positive; *n*, number; %, percentage; *p*, *p* value; Chi-square test for trend.

**Table 5:** The relationship between the severity of cervical inflammation and cytological abnormalities and HPV positivity.

Cervical Inflammation	Smear Cytology Diagnosis		HPV	
	(−) %(*n*)	(+) %(*n*)	(−) %(*n*)	(+) %(*n*)
Minimal	91.1(133)	8.9(13)	87.7(71)	12.3(10)
Moderate	85.6(202)	14.4(34)	82.1(92)	17.9(20)
Severe	82.9(63)	17.1(13)	92.0(23)	8.0(2)
Test value	3.683		2.146	
*p*	0.159		0.342	

Note: HPV, human papillomavirus; *n*, number; %, percentage; (−), benign; (+), Low-grade squamous intraepithelial and above; *p*, *p* value Chi-square test for trend.

**Table 6:** Multivariable logistic regression analysis for abnormal cytology and HPV positivity.

Variable	Abnormal Cytology aOR	95% CI	*p* Value	HPV Positivity aOR	95% CI	*p* Value
Age	0.983	0.945–1.021	0.373	1.047	0.980–1.119	0.175
Smoking, yes vs. no	0.671	0.371–1.214	0.187	0.710	0.292–1.723	0.448
Parity, overall	—	—	0.742	—	—	0.173
Vaginal discharge, yes vs. no	0.877	0.396–1.942	0.746	2.198	0.600–8.059	0.235
Symptom recurrence frequency, overall	—	—	0.285	—	—	0.425
Candida-like cytomorphological findings, yes vs. no	0.654	0.137–3.121	0.595	3.752	0.495–28.440	0.201
Cytological findings suggestive of bacterial vaginosis, yes vs. no	0.473	0.060–3.741	0.478	0.776	0.063–9.622	0.843
Cervical inflammation severity, overall	—	—	0.309	—	—	0.204
Moderate inflammation vs. minimal inflammation	1.670	0.830–3.361	0.151	1.875	0.764–4.601	0.170
Severe inflammation vs. minimal inflammation	1.764	0.739–4.213	0.201	0.597	0.104–3.436	0.563
Symptom burden, overall	—	—	0.977	—	—	0.238

aOR, adjusted odds ratio; CI, confidence interval; HPV, human papillomavirus; BV, bacterial vaginosis. Binary logistic regression models were adjusted for age, smoking status, parity, vaginal discharge, symptom recurrence frequency, Candida positivity, bacterial vaginosis, cervical inflammation severity, and symptom burden. The Hosmer–Lemeshow test indicated no evidence of poor model fit for abnormal cytology (*p* = 0.634) or HPV positivity (*p* = 0.318). Nagelkerke R^2^ values were 0.052 and 0.162, respectively. For variables with more than two categories, overall *p* values are presented. HPV-related analyses were performed among participants with available HPV results (*n* = 218).

## Data Availability

The original contributions presented in this study are included in the article Further inquiries can be directed to the corresponding author(s).
